# Psychological factors of suspect coronary microvascular dysfunction in patients undergoing SPECT imaging

**DOI:** 10.1007/s12350-020-02360-5

**Published:** 2020-10-06

**Authors:** Maria T. Bekendam, Ilse A. C. Vermeltfoort, Willem J. Kop, Jos W. Widdershoven, Paula M. C. Mommersteeg

**Affiliations:** 1 Center of Research on Psychology in Somatic diseases (CoRPS), Department of Medical and Clinical Psychology, Tilburg University, Warandelaan 2, P.O. Box 90153, 5000 LE Tilburg, The Netherlands; 2 Department of Nuclear Medicine, Institute Verbeeten, Tilburg, The Netherlands; 3 Department of Cardiology, Elizabeth-TweeSteden Hospital, Tilburg, The Netherlands

**Keywords:** Cardiac stress testing, Emotions, Myocardial ischemia, Psychological factors, Suspect CMD

## Abstract

**Background:**

Patients with myocardial ischemia in the absence of obstructive coronary artery disease (CAD) often experience anginal complaints and are at risk of cardiac events. Stress-related psychological factors and acute negative emotions might play a role in these patients with suspect coronary microvascular dysfunction (CMD).

**Methods and Results:**

295 Patients (66.9 ± 8.7 years, 46% women) undergoing myocardial perfusion single-photon-emission computed tomography (MPI-SPECT), were divided as follows: (1) a non-ischemic reference group (*n *= 136); (2) patients without inducible ischemia, but with a history of CAD (*n *= 62); (3) ischemia and documented CAD (*n *= 52); and (4) ischemia and suspect CMD (*n *= 45). These four groups were compared with regard to psychological factors and acute emotions. Results revealed no differences between the groups in psychological factors (all *P* > .646, all effect sizes d < .015). State sadness was higher for patients with suspect CMD (16%) versus the other groups (*P *= .029). The groups did not differ in the association of psychological factors or emotions with anginal complaints (all *P* values > .448).

**Conclusion:**

Suspect CMD was not associated with more negative psychological factors compared to other groups. State sadness was significantly higher for patients with suspect CMD, whereas no differences in state anxiety and other psychological factors were found.

**Electronic supplementary material:**

The online version of this article (10.1007/s12350-020-02360-5) contains supplementary material, which is available to authorized users.

## Introduction

The presence of myocardial ischemia in the absence of obstructive CAD (non-obstructive CAD; coronary stenosis < 50%) is not as benign as previously assumed and is associated with elevated cardiovascular risk^1^,^2^ such as recurrent diagnostic testing, poor quality of life, hospitalization, and long-term adverse cardiac events^3^–^5^. In addition, in 44% of patients with non-obstructive CAD, chest pain symptoms are still reported 3 months after CAG, and chest pain is related to increased psychological distress^6^. In the present study, we aim to examine psychological factors and acute emotions in patients referred for myocardial perfusion imaging to detect myocardial ischemia.

Coronary microvascular dysfunction (CMD) involves narrowing or endothelial dysfunctioning of the small coronary arteries resulting in myocardial ischemia^4^,^7^–^9^. CMD is not easily detected by routine CAG. It is hypothesized that patients with non-obstructive CAD who continue to report cardiac symptoms may have suspect underlying CMD^4^. In the present study, suspect CMD is operationalized as the presence of myocardial ischemia observed using myocardial perfusion imaging (MPI), while obstructive CAD has been ruled out based on coronary angiography. This operationalization is consistent with the suspect microvascular angina definition of Ong^9^. There is a need to examine the role of psychological factors and acute emotions in the CMD group, given the prevalence of distress associated with recurrent symptoms such as anginal chest pain^6^.

A growing number of studies has established links between anxiety, higher rates of cardiac risk factors, and increased risk of cardiovascular events^10^–^13^. Anxiety has been found to be associated with persistent chest pain, more severe cardiac symptoms, and lower rates of obstructive CAD overall among a female cohort with ischemia and a low rate of obstructive CAD^14^–^16^. In addition to anxiety, depression, anger and personality are among the most well-documented psychological risk factors associated with ischemic heart disease^12^,^17^–^19^.

The association between acute emotions and ischemia during MPI is likely^20^–^25^, but evidence to date is based on self-reported emotions only. The unique aspect of this study is the measurement of acute emotions using facial expression recognition software during cardiac stress testing as part of MPI^26^. It is unknown whether acute negative emotions measured during cardiac stress testing are associated with the presence or absence of anginal complaints reported during cardiac stress testing in patients with (suspect) CMD.

The following three comparison groups will be examined: (1) patients without ischemia and no (history of) obstructive CAD, (non-ischemic reference group), (2) patients with no inducible myocardial ischemia but clinical history of CAD (history of CAD), and (3) patients with ischemia and (history of) obstructive CAD (ischemic CAD). It is hypothesized that (1) patients with suspect CMD are characterized by significantly more psychological factors that are associated with ischemic heart disease (general anxiety, depressive symptoms, a lower well-being and Type D personality) than the non-ischemic reference group, and (2) that patients with suspect CMD display significantly more expressions of negative acute emotions (anxiety and sadness) during cardiac stress testing compared to the other three comparison groups. In addition, it is hypothesized that the association between psychological factors and emotional states during cardiac stress testing with anginal complaints is stronger in patients with suspect CMD compared to patients with CAD.

## Methods

### Patients

The study sample consisted of 295 patients undergoing a stress/rest myocardial perfusion imaging single-photon-emission computed tomography (MPI-SPECT, henceforth referred to as MPI) protocol, using exercise stress testing (*N* = 88) or pharmacologically induced stress testing (*N* = 207). Patient enrollment took place between January 2017 and December 2018 at the Institute Verbeeten in Tilburg, The Netherlands. Inclusion criteria were: (1) referral for the MPI protocol with adenosine stress testing or exercise stress testing; (2) ability to fill out questionnaires; and (3) sufficient knowledge of the Dutch language. There were no exclusion criteria. Reasons for referral to the Institute Verbeeten were categorized into ‘new-onset cardiac symptoms’ (*N* = 181) or ‘returning cardiac symptoms’ (*N* = 114). All included patients in this study underwent the protocol as described below with MPI images obtained at rest and following cardiac stress testing. Cardiac stress testing entails both pharmacological adenosine stress testing, and exercise stress testing on an exercise bicycle; both will be referred to as cardiac stress testing in the remainder of this paper. During cardiac stress testing, patients’ facial expressions were video-recorded using a webcam (Logitech C920 – HD Pro) attached to the stationary exercise bicycle (GE Healthcare, Ergometer ebike comfort 162202, Freiburg, Germany). Sociodemographic and psychological data were collected before, during, and after cardiac stress testing (see below for details). All included patients provided written informed consent and the study was approved by the regional Medical Ethics Committee (METC Brabant, Protocol number: NL56707.028.16).

#### Assessment of myocardial ischemia

The MPI protocol entailed two days, with a rest imaging day and a stress imaging day (adenosine stress testing or exercise stress testing). Beforehand, patients received instructions to refrain from consuming caffeine-containing beverages for 24 hours before each protocol day. On the rest imaging day, patients received a ^99m^Tc-tetrofosmin injection (dosage: 370 MBq), followed by a rest period of 45 minutes. Subsequently, they underwent myocardial perfusion imaging. On the stress imaging day, patients performed cardiac stress testing, either pharmacologically by intravenous adenosine injection (140 mcg/kg/minute for 5 minutes) or by exercise (i.e., bicycling to maximum exertion using the modified Bruce protocol). According to standard clinical protocol, the adenosine cardiac stress testing also involved low intensity cycling to limit adenosine side effects and reduce extracardiac activity^27^. During the adenosine protocol, the ^99m^Tc-tetrofosmin injection was at 2 minutes after adenosine administration. For the exercise protocol, the injection was at peak exercise, more specifically at 85% of the maximum heartrate (0.85*(220-patient age)).

### Perfusion Imaging

Image acquisition was performed using a hybrid dual-headed gated IQ SPECT/CT system (Symbia T, Siemens Medical Solutions AG) equipped with multifocal collimators (SmartzoomTM) of 128 × 128 matrix size and zoom factor of 1. Acquired data were then reconstructed using an iterative reconstruction. Attenuation correction was applied using a patient dedicated low-dose CT-derived mu map. A symmetric 15% window was centered at 140 keV, with three-lead electric cardiographic monitoring. Perfusion images were inspected by qualified staff before interpretation by experienced nuclear physicians.

### Myocardial Ischemia and Defining Groups

For the interpretation of perfusion images, bull’s eye generation and visual analysis using a 17-segment model^28^, was performed by two experienced observers. Semiquantitative analyses of perfusion were performed with QPS software from Cedars-Sinai Medical Center. Quantitative summed rest scores (SRS), summed stress scores (SSS), and summed difference scores (SDS) were tabulated. Myocardial perfusion was graded on a 0-4 scale: 0 = normal, 1 = equivocal, 2 = moderate, 3 = severe perfusion defect, and 4 = very severe perfusion defect. Interpretation of the myocardial perfusion imaging was assessed by visual analysis as recommended by the American Society of Nuclear Cardiology (ASNC)^29^. Both AC and no-AC images were reviewed during interpretation. Myocardial ischemia was semi-quantitatively defined as SDS ≥ 2^30^.

For the groups with present ischemia, records of CAG or CTCA following or preceding myocardial perfusion imaging were checked within a maximum period of 6 months after study inclusion. If significant coronary obstructions (≥ 50% of the left main coronary artery, or ≥ 50% in other epicardial coronary arteries) were found, patients were grouped into the present ischemia and obstructive CAD group, in short: ‘ischemic CAD’ group. Presence of ischemia, but no significant obstructions (< 50% or otherwise not in need of cardiac intervention) resulted in assignment to the suspect CMD group. In total four groups were created: a non-ischemic reference group with no ischemia and no cardiac history (non-ischemic reference group, *N* = 136), a group with no present ischemia and no need for subsequent diagnostic CAG, but who in the past were treated for obstructive CAD (history of CAD: *N* = 62), the group with present ischemia and obstructive CAD (ischemic CAD group, *N* = 52), and the group with present ischemia, but no obstructive CAD (suspect CMD group, *N* = 45).

### Video Recordings During Cardiac Stress Testing to Measure State Anxiety and State Sadness

Patients were video-recorded during cardiac stress testing for later analysis of facial expressions. Cardiac stress testing for each patient was divided into four consecutive time blocks for the purpose of analysis: ‘baseline’, ‘exertion’, ‘maximal (max.) exertion’, and ‘recovery’. The ‘baseline’ time block comprised video recording the first two minutes while the patient was already on the exercise bicycle, just before cardiac stress testing started. ‘Exertion’ comprised video recording the two minutes (adenosine stress testing), or first several minutes (exercise stress testing) of cardiac stress testing, before actual administration of the radioactive tracer. ‘Max. exertion’ entailed the one minute video recording at two minutes after adenosine administration (adenosine stress testing), or several minutes of exertion at peak heart rate (exercise stress testing). Finally, ‘recovery’ comprised a one minute recording of coming to a stop (adenosine stress testing), or slowly coming to a stop (exercise stress testing).

### Anginal Complaints and Symptoms During Cardiac Stress Testing

During cardiac stress testing (both protocols) patients were asked whether they experienced anginal complaints (anginal chest pain present/absent) similar to their “typical” cardiac symptoms. This dichotomous measure used in the present study was based on MPI reportings by the nuclear physician present during cardiac stress testing.

Patients were also asked to indicate specific symptoms during cardiac stress testing. These symptoms included shortness of breath, dizziness, nausea, fatigue, and hot flushes. Patients were also asked to indicate whether they felt anxious or tense. A dichotomous (present/absent) measure was used in analyses.

### Facial Expression Analysis of Acute Emotions

The facial expression recognition software package FaceReader 7.0^31^ was used for analyzing the video recordings made prior to and during cardiac stress testing. Analyses were conducted at the GO-LAB (Gedragsfysiologisch Onderzoekslaboratorium; Behavioral-physiological research laboratory) of Tilburg University, The Netherlands. The FaceReader software broadly works as follows: it finds a patient’s face and creates a 3D Active Appearance Model of the face^32^. FaceReader additionally uses artificial network analysis in order to recognize patterns in the face^33^. This combination of Active Appearance Model and pattern recognition is used to compute scores of intensity of facial expressions on a continuous scale from 0 to 1 (0%-100% in a specific time block), yielding the percentage of intensity of the acute emotions anxiety and sadness. In each video, 15 frames per second are analyzed^34^, and a mean percentage of each acute emotion during the four consecutive cardiac stress testing time blocks was reported. Facial expression analyses of the present study were based on all available frames obtained during the four time blocks. The mean number of recorded frames per time block were: ‘baseline’: 2367.8 ± 1640.4, ‘exertion’: 2436.9 ± 1781, ‘max exertion’: 1818.8 ± 913.8, ‘recovery’: 1107 ± 574.7. Before each individual video recording started, the patient’s face was calibrated over a consecutive two second period (30 frames out of the 5-second calibration phase), in which FaceReader corrects for individual facial features that could result in biases towards a certain facial expression.

### Questionnaires Addressing Psychological Factors

Patients were asked to fill out several (standardized) questionnaires to obtain information on psychological factors and sociodemographics.

#### General anxiety

The generalized anxiety disorder (GAD-7) scale is a validated clinical screening measure used for assessing generalized anxiety disorder. The GAD-7 consists of seven items that are answered on a four-point scale from 0 (“not at all”) to 3 (“nearly every day”). The GAD-7 has been validated in the general population^35^, and the scale has a high internal consistency (Cronbach’s alpha = .89). For present analyses, the continuous score of the GAD-7 was used, with a higher score indicating more generalized anxiety symptoms.

#### Depressive symptoms

Depressive symptoms were assessed with the PHQ-9 questionnaire. Each of the PHQ-9 items corresponds to one of the nine DSM criteria for diagnosis of major depression. Patients are asked to indicate the presence of each of the items, representing a depressive symptom (0 = not at all, 1 = several days, 2 = more than half of the days, 3 = nearly every day). The earlier reported internal consistency for the PHQ-9 is high (Cronbach’s alpha = .89)^36^. The continuous PHQ-9 measure was used in this study, with a higher score indicating more depressive symptoms.

#### Well-being

Dimensions of positive mental health were assessed with the Mental Health Continuum-short form (MHC-SF), a 14-item questionnaire measuring well-being. The different dimensions of well-being the MHC-SF measures are emotional well-being (3 items), social well-being (5 items), and psychological well-being (6 items). The MHC-SF has been validated in earlier studies^37^ reporting a high internal consistency (Cronbach’s alpha = .89). The continuous well-being score (higher score indicating higher well-being) was used in the present study.

#### Type D personality

Type D personality was assessed using the Type D Scale-14 (DS-14). Patients rated their personality on a 5-point Likert scale ranging from 0 (false) to 4 (true). The negative affect (NA) and social inhibition (SI) scales can be scored as continuous variables (range, 0-28) to assess these personality traits in their own right. The internal consistency of the NA and SI scales is high (Cronbach’s alpha NA = .88; SI = .86)^38^.

### Sociodemographic Information, Cardiovascular Risk Factors, Medication, and Cardiac History

Sociodemographic information such as gender, age, BMI, smoking, and cardiovascular risk factors such as hypertension, hypercholesterolemia, diabetes mellitus, and familial risk of cardiovascular disease (first degree family members with cardiac event) were obtained using questionnaires and electronic hospital records, respectively. Hypertension, hypercholesterolemia, and diabetes mellitus were based on hospital record information on presence of the risk factor, or use of medication for hypertension (ACE/ARB inhibitors, beta-adrenergic blocking agents, calcium inhibitors), cholesterol lowering medication (statins, fibrates, or other), or diabetes medication, respectively. Antithrombotic medication comprised anticoagulants and antiplatelets. Antiplatelets (aspirin) were further specified as acetylsalicylic acid Ascal (ATC: B01AC06/08/30) or a combination with acetylsalicylic acid. Both antidepressant and anxiolytic medication (ATC: N05B/N05C) use were obtained. Cardiac history included previous myocardial infarction (MI), percutaneous coronary intervention (PCI), and coronary artery bypass grafting (CABG).

### Statistical Analyses

Data were presented as means ± standard deviation (SD) for continuous variables and frequencies and percentages for categorical variables, stratified by group and presence/absence of anginal complaints as reported during cardiac stress testing. Group comparisons for categorical variables were examined using Chi^2^ tests and continuous variables using one-way ANOVA tests. Logistic regression analyses were performed to explore whether general anxiety, depressive symptoms, well-being, Type D personality, state anxiety, and state sadness were associated with presence of anginal complaints during cardiac stress testing, whether this was significantly different for the four groups, and if interactions were observed. The four groups were separately included as dummy variables with each dummy group compared to the other three groups. General anxiety, depressive symptoms, well-being, Type D personality, state anxiety, and state sadness were explored in separate models. The psychological factors and acute emotions, the four groups (dummy coded), and interactions between the groups and the psychological factors and acute emotions were entered in the each model, adjusted for age, gender, and LVEF at rest (considering the differences of LVEF associated with inducible ischemia^39^). Age, gender, and LVEF were entered in model 1. In model 2, the four groups and the psychological factors (in separate models for each measure; general anxiety, depressive symptoms, well-being, Type D personality, state anxiety, and state sadness) total scores were added. In model 3, the interaction term of the group dummy variable with the psychological factor was added. Odds ratios (OR) with 95% confidence intervals (CIs) are reported. Statistical analyses were performed using SPSS version 24.0 (SPSS Inc., Chicago, Illinois), two-sided p values are reported, and statistical significance was set at *P* < 0.05.

## Results

### Patient Characteristics and Cardiac Symptoms During Cardiac Stress Testing

Table 1 displays the baseline characteristics of the study sample. The mean age was 66.9 ± 8.7 years, and 46% (*N *= 135) were women. There were significant gender differences between the four groups; women were more prevalent in the suspect CMD group (56%) and the reference group (59%), whereas men were significantly more often represented in the ischemic CAD group (79%) and the history of CAD group (69%) than women (*P* < .001).

**Table 1:** Patient characteristics stratified by group

Baseline characteristic	Reference^a^	History of CAD^b^	Ischemic CAD^c^	Suspect CMD^d^	*P* value
Group	136 (46%)	62 (21%)	52 (18%)	45 (15%)	
Gender (women)	**80 (59%)**	**19 (31%)**	**11 (21%)**	**25 (56%)**	**<.001**
Age (years)	66.7 ± 8.0	66.3 ± 9.0	67.8 ± 9.0	67.0 ± 10.1	.817
College education (yes)	91 (68%)	34 (55%)	33 (64%)	24 (59%)	.319
Partner (yes)	104 (77%)	54 (87%)	43 (84%)	37 (86%)	.266
Cardiac risk factors					
BMI (kg/m^2^)	28.2 ± 5.9	27.7 ± 4.6	29.8 ± 7.1	28.2 ± 4.1	.254
Obese (BMI > 30)	38 (30%)	14 (24%)	20 (39%)	12 (30%)	.365
Smoking (yes)	16 (12%)	12 (19%)	8 (15%)	6 (14%)	.586
Hypertension	63 (53%)	21 (40%)	28 (61%)	17 (41%)	.110
Hypercholesterolemia	**73 (54%)**	**58 (94%)**	**44 (85%)**	**30 (67%)**	**<.001**
Diabetes Mellitus	48 (35%)	18 (29%)	24 (46%)	17 (38%)	.295
Cardiac familial risk (first degree family members with cardiac event)	75 (56%)	40 (66%)	31 (61%)	23 (56%)	.614
Reasons for referral					
New-onset cardiac symptoms	88 (65%)	34 (56%)	28 (55%)	30 (67%)	.409
Returning cardiac symptoms	48 (35%)	27 (44%)	23 (45%)	15 (33%)	
Medication					
Anticoagulants	**68 (50%)**	**61 (98%)**	**48 (92%)**	**34 (76%)**	**<.001**
Of which aspirin	**41 (30%)**	**53 (86%)**	**38 (71%)**	**29 (64%)**	**<.001**
ACE-ARB inhibitors	*59 (43%)*	*36 (58%)*	*32 (62%)*	*19 (42%)*	*.050*
Beta-blocker	**58 (43%)**	**39 (63%)**	**31 (60%)**	**25 (56%)**	**.027**
Calcium inhibitors	**28 (21%)**	**20 (32%)**	**21 (40%)**	**12 (27%)**	**.040**
Diuretics	32 (24%)	16 (26%)	13 (25%)	9 (20%)	.909
Cholesterol lowering meds	**57 (42%)**	**57 (92%)**	**44 (85%)**	**25 (56%)**	**<.001**
Nitrates	**24 (18%)**	**31 (50%)**	**27 (52%)**	**14 (31%)**	**<.001**
Antidepressants	17 (13%)	4 (7%)	2 (4%)	5 (11%)	.244
Anxiolytic medication*	12 (9%)	2 (3%)	3 (6%)	5 (11%)	.382

*Benzodiazepines; Data presented as mean ± standard deviation or number (%)

*BMI* body mass index; *CAD* coronary artery disease; *CMD* coronary microvascular dysfunction

^a^No ischemia /no cardiac history

^b^No ischemia/history of obstructive CAD

^c^Ischemia, obstructive CAD

^d^Ischemia, non-obstructive CAD

The prevalence of hypercholesterolemia was highest in the history of CAD group (94% (history of CAD group) vs. 85% (ischemic CAD), 67% (suspect CMD), and 54% (non-ischemic reference group), *P* < .001). No other significant differences were found for traditional cardiac risk factors between the four groups (all *P* values > .110, all Cramer’s V < .153, and all *η*^2^ < .014). Medication use was structurally higher in the group with a history of CAD and ischemic CAD.

Table 2 displays cardiac symptoms during cardiac stress testing and MPI measures. No significant differences were found for self-reported physical symptoms during cardiac stress testing (all *P* values > .202, all Cramer’s V < .133). MPI measures (SRS, SSS, SDS) were significantly different between the suspect CMD and ischemic CAD group with SRS, SSS, and SDS being higher for the ischemic CAD group (SRS: 3.75; SSS: 8.37; SDS: 4.88) than for the suspect CMD group (SRS: 1.98; SSS: 5.22; SDS: 4.88, *P* < .001). LVEF during both rest and stress was higher for the suspect CMD group (rest: 60.2% ± 11.3%; stress: 57.0% ± 12.6%) compared to the history of CAD (rest: 55.0% ± 14.0%, *P* < .001; stress: 53.9% ± 13.7%, *P* = .614) and the ischemic CAD group (rest: 55.5% ± 11.9%, *P* = .002; stress: 53.9% ± 12.2%, *P* = .639) (Table 2).

**Table 2:** Cardiac symptoms during cardiac stress testing and MPI and LVEF measures stratified for groups

	Reference^a^ (*n* = 136)	History of CAD^b^ (*n *= 62)	Ischemic CAD^c^ (*n *= 52)	Suspect CMD^d^ (*n *= 45)	*P* value
Type of cardiac stress testing					
Adenosine (versus cycling)	87 (64%)	42 (68%)	35 (67%)	37 (82%)	.151
Symptoms during cardiac stress testing					
Anginal complaints	40 (29%)	23 (37%)	20 (39%)	14 (31%)	.564
Shortness of breath	60 (52%)	32 (56%)	19 (40%)	17 (43%)	.313
Dizziness	43 (37%)	13 (23%)	18 (38%)	16 (40%)	.202
Nausea	16 (14%)	10 (18%)	6 (13%)	5 (13%)	.870
Fatigue	37 (32%)	18 (32%)	13 (28%)	14 (35%)	.906
Flushing	52 (45%)	25 (44%)	17 (36%)	13 (33%)	.469
MPI measures					
Summed rest score (SRS)	**0.8 ± 2.5**	**2.8 ± 5.5**	**3.8 ± 5.7**	**2.0 ± 3.3**	**<.001**
Summed stress score (SSS)	**1.2 ± 2.7**	**2.7 ± 5.4**	**8.4 ± 8.1**	**5.2 ± 4.6**	**<.001**
Summed difference score (SDS)	**0.4 ± 0.6**	**0.4 ± 1.3**	**4.9 ± 4.4**	**3.5 ± 2.2**	**<.001**
LVEF					
LVEF (rest) %	**63.3 ± 12.6**	**55.0 ± 14.0**	**55.5 ± 11.9**	**60.2 ± 11.3**	**<.001**
LVEF (stress) %	**62.4 ± 12.0**	**53.9 ± 13.7**	**53.9 ± 12.2**	**57.0 ± 12.6**	**<.001**

Data presented as mean ± standard deviation or number (%)

*CAD* coronary artery disease; *CMD* coronary microvascular dysfunction; *MI* myocardial infarction; *PCI* percutaneous coronary intervention; *CABG* coronary artery bypass grafting; *CAD* coronary artery disease; *NA* negative affectivity; *SI* social inhibition

^a^No ischemia /no cardiac history

^b^No ischemia/history of obstructive CAD

^c^Ischemia, obstructive CAD

^d^Ischemia, non-obstructive CAD

Hemodynamic and severity of ischemia comparisons stratified for the adenosine and cycling cardiac stress test protocols are presented in Supplemental Table S1.

### Psychological Factors and Acute Emotions in Patients with Suspect CMD

Group differences for psychological factors and acute emotions during cardiac stress testing are shown in Table 3. No significant differences were found for general anxiety, or any of the other psychological factors and mental health status between the four groups (all *η*^2^ < .015, all *P* values > .078).

**Table 3:** Psychological factors and acute emotions during cardiac stress testing stratified for groups

	Reference^a^ (*n* = 136)	History of CAD^b^ (*n *= 62)	Ischemic CAD^c^ (*n *= 52)	Suspect CMD^d^ (*n *= 45)	*P* value
Psychological factors					
General anxiety [GAD-7]	5.9 ± 5.7	5.3 ± 5.5	4.8 ± 4.9	5.5 ± 4.8	.646
Depressive symptoms [PHQ-9]	6.2 ± 5.8	6.0 ± 4.7	5.6 ± 5.2	5.9 ± 5.1	.941
Well-being [MHC-SF]	43.0 ± 12.4	43.7 ± 11.7	44.2 ± 15.3	41.7 ± 12.7	.825
Type D personality [DS-14]					
Negative affectivity	10.1 ± 6.3	9.3 ± 5.4	9.2 ± 6.0	10.1 ± 5.8	.717
Social inhibition	8.4 ± 5.8	9.5 ± 5.9	8.6 ± 6.5	8.3 ± 5.7	.665
Acute emotions during cardiac stress testing					
State anxiety score [mean]					
Anxiety baseline	2.9 ± 2.8	2.9 ± 2.4	2.6 ± 2.2	3.2 ± 2.9	.811
Anxiety max. exertion	3.1 ± 4.2	2.9 ± 3.0	3.6 ± 4.6	2.9 ± 3.1	.816
State sadness score [mean]					
Sadness baseline	9.1 ± 7.1	8.6 ± 8.5	9.3 ± 6.9	9.5 ± 6.0	.949
Sadness max. exertion	**9.7 ± 9.7**	**13.8 ± 17.2**	**10.9 ± 10.8**	**15.9 ± 12.2**	**.029**
Self-Reported tension/anxious	**31 (27%)**	**3 (5%)**	**3 (6%)**	**7 (18%)**	**.001**

Data presented as mean ± standard deviation or number (%)

*CAD* coronary artery disease; *CMD* coronary microvascular dysfunction; *NA* negative affectivity; *SI* social inhibition

^a^No ischemia /no cardiac history

^b^No ischemia/history of obstructive CAD

^c^Ischemia, obstructive CAD

^d^Ischemia, non-obstructive CAD

Regarding emotional expression during cardiac stress testing, no significant differences between the groups were found for the facial expression of state anxiety (all *η*^2^ < .040; all *P* values > .665). In patients with suspect CMD only state sadness during maximal exertion was significantly higher than for the other three groups (Table 3). Emotional expression during cardiac stress testing was stratified for cardiac stress testing protocol (Supplemental Table S2). State sadness during maximal exertion was highest in the CMD (18.91 ± 19.10) and the history of CAD group (19.22 ± 24.76) in the exercise protocol, but not in the adenosine protocol. With regard to self-reported acute emotions, it was found that patients in the non-ischemic reference group and the suspect CMD group more often reported tension/anxious symptoms than the two three groups (27% (non-ischemic reference group), and 18% (suspect CMD) vs. 6% (ischemic CAD), and 5% (history of CAD), *P* = .001, Cramer’s *V* = .257).

### Association of Psychological Factors and Emotional States with Anginal Complaints

The four groups did not differ significantly in the association between general anxiety or other psychological factors (depressive symptoms, well-being, and Type D personality) with anginal complaints during cardiac stress testing (ORs interaction psychological variable x group < 2.73, all *P* values > .056). Similarly, acute emotions during cardiac stress testing (anxiety and sadness) were not differentially associated with anginal complaints (ORs interaction acute emotions x group < 1.03, all *P* values > .378) (data not shown).

## Discussion

The present study aimed to examine psychological factors, acute emotions, and cardiac symptoms in patients with suspect CMD. Patients with suspect CMD were compared to patients with ischemic CAD, patients with a history of CAD, and a non-ischemic reference group with no ischemia and no cardiac history. The psychological factors general anxiety, depressive symptoms, well-being, and Type D personality did not significantly differ between patients with suspect CMD and the three other groups. However, patients with suspect CMD showed significantly more facial expression of state sadness compared to the other groups during the maximal exertion of cardiac stress testing. Explorative analyses showed that the association of general anxiety, depressive symptoms, well-being, Type D personality, state anxiety, and state sadness with anginal complaints reported during cardiac stress testing in patients with suspect CMD was not significantly different from the other three groups.

There is a vast number of studies conducted on acute emotions, but mostly in combination with a different focus rather than exercise-induced myocardial ischemia and suspect CMD. For example, mental stress-induced myocardial ischemia (MSIMI) is more common in patients with exercise-induced ischemia than in patients without exercise-induced ischemia^40^ and is associated with depression rather than state anxiety^41^,^42^. The relatively high prevalence of MSIMI among women may be attributable to pathophysiological mechanisms such as CMD^40^. In our sample, women were indeed significantly more present in the suspect CMD group than were men. Further, our findings showed that state sadness was associated with suspect CMD and this finding could be relevant to the assessment of depression in patients with CMD. However, no association with depressive symptoms or anxiety was observed in our sample. Furthermore, it was found that triggers of acute coronary events (ACS; myocardial infarction, unstable angina, or sudden cardiac death) include acute emotional reactions such as state anxiety^43^, but we did not observe similar findings for anginal complaints. It should be noted that state-like triggers might be amplified by trait-like factors such as personality and generalized anxiety^43^, although neither were associated with suspect CMD in the present study.

These findings are consistent with a study where patients with normal coronary arteries and anginal complaints, with a higher female prevalence compared to males, and patients with CAD and anginal complaints did not differ on any psychological measure including stress, anxiety, and depression^44^. However, a follow-up study showed that, among patients with anginal complaints but normal coronary arteries, levels of psychological morbidity were significantly higher one year and eleven years after baseline than in patients with CAD^45^. This suggests that the association of anginal complaints with psychological factors in the group with chest pain and normal coronary arteries might become apparent after considerable time has passed. In contrast, another study found that patients with anginal complaints, myocardial ischemia, and non-obstructive coronary arteries suffered significantly higher levels of generalized anxiety than CAD patients^46^. Furthermore, different studies showed that patients with ischemia, microvascular abnormalities, and no coronary stenosis have a lower threshold for chest pain than patients with CAD^47^, but this was not confirmed in the present study. Most studies concerning psychological factors discussed here are based on patients with cardiac syndrome X, a diagnosis that is deemed inadequate^48^ but has considerable overlap with more recent definitions of suspect CMD, including the definition by Ong and colleagues used in the present study^9^.

Different definitions of patients with suspect myocardial dysfunction or microvascular angina persist and diagnostic inclusion or exclusion criteria are broad or not well defined. The overall definition of cardiac syndrome X constituted: 1. anginal episodes, 2. evidence of myocardial ischemia, 3. normal, or near normal coronary arteries at CAG^49^, and does not vastly differ from the definition used by Ong and colleagues^9^, which states: 1. symptoms suggestive of myocardial ischemia (e.g., angina), 2. absence of obstructive CAD, 3. objective evidence of myocardial ischemia, 4. impaired coronary microvascular function (e.g., reduced coronary blood flow reserve). The latter definition contains the addition of reduced coronary blood flow reserve, which is also found in the cardiac syndrome X definition by the Lanza review^48^. Exclusion criteria for the different definitions, whether it be a definition for cardiac syndrome X or suspect CMD, are unclear. In a review by Suzuki et al (2015), the definition of microvascular angina is extended with the exclusion criterion of any other specific cardiac disease such as cardiomyopathy, hypertensive heart disease, and valvular heart disease^50^. This is consistent with an earlier cardiac syndrome X definition^48^, but the definition by Ong and colleagues does not specifically mention particular exclusion criteria^9^. As a consequence, the sample used in the present study can be argued to be heterogeneous, despite adhering to one of the most recent suspect CMD definitions^9^. The lack of consensus on exclusion criteria and the heterogeneous character of patients with myocardial ischemia in the absence of significant coronary obstructions is noteworthy and has to be taken into account in interpreting and conducting future studies in this group.

### Limitations and Strengths

The present study has some further limitations and strengths that should be taken into account. Limitations include the relatively small suspect CMD patient group sample and the cross-sectional design. Reliance on SPECT to document myocardial ischemia may have resulted in an underdetection of CMD and that more sensitive methods (e.g., PET) could have detected less severe CMD. Future studies are needed to examine psychological factors in patients with CMD as determined by a combination of coronary flow reserve (CFR) and fractional flow reserve (FFR). The incorporation of a validated questionnaire to measure general anxiety, depressive symptoms, well-being, and Type D personality can be considered a strength of this study. A characterizing aspect is the measurement of state anxiety and state sadness with facial expression recognition software during cardiac stress testing. Although reported findings were not statistically significant, it is a validated^31^ measure of facial expression of acute emotions.

Research on patients with suspect CMD and the association with psychological factors and acute emotions is still in an early stage. This study is one of the first to incorporate psychological factors and acute emotions, measured with facial expression software, into suspect CMD research. Although our sample is based on recent suspect CMD criteria, it is still a rather broad patient group, and further (follow-up) studies are needed to differentiate the effect of diverse psychological factors and acute emotions in patients with suspect CMD.

## Conclusion

The present findings indicate that psychological factors measured during cardiac stress testing are not significantly different for patients with suspect CMD compared to patients with (history of) CAD, and a non-ischemic reference group with no ischemia and no cardiac history. State sadness was significantly higher for patients with suspect CMD. The relation between psychological factors and cardiac patients is a complicated one, which needs further investigation in order to shed more light on patients who keep experiencing debilitating complaints but who cannot receive optimal treatment.

## New Knowledge Gained

Our data indicate that psychological factors measured during cardiac stress testing are not significantly different for patients with suspect CMD compared to patients with (history of) CAD. Patients with suspect CMD are not characterized by more (trait) psychological factors, but state sadness, however, was significantly higher for patients with CMD during cardiac stress testing.

## Electronic supplementary material

Below is the link to the electronic supplementary material.

(PDF 726 kb)

(PDF 686 kb)

(PPTX 194 kb)

(DOCX 12 kb)

## Abbreviations

CAD: Coronary artery disease
CAG: Coronary angiography
CMD: Coronary microvascular dysfunction
LVEF: left ventricular ejection fraction
MPI: Myocardial perfusion imaging
SDS: Summed difference score
SRS: Summed rest score
SSS: Summed stress score
