# Supplementary material for: Psychological factors of suspect coronary microvascular dysfunction in patients undergoing SPECT imaging
Source: J Nucl Cardiol. 2020 Oct 6;29(2):768–78. doi: 10.1007/s12350-020-02360-5 (PMC8993740; doi:10.1007/s12350-020-02360-5)
Supplement: Supplementary file 1 — (PDF 726 kb) [file 12350_2020_2360_MOESM1_ESM.pdf]

Supplemental Table S1. Hemodynamics during cardiac stress testing for the adenosine and exercise protocol

	**Adenosine protocol (n = 203)**					**Exercise protocol (n = 88)**				
	Reference[1] (n=87)	History of CAD[2] (n=42)	Ischemic CAD[3] (n=35)	Suspect CMD[4] (n=37)	p-value	Reference[1] (n=44)	History of CAD[2] (n=20)	Ischemic CAD[3] (n=17)	Suspect CMD[4] (n=7)	p-value
LVEF										
LVEF rest (%)	**62.2±12.6**	**52.6±14.6**	**55.7±11.7**	**59.3±11.8**	**.001**	*64.8±12.9*	*59.5±12.0*	*55.2±12.7*	*63.3±8.04*	*.062*
LVEF stress (%)	**60.1±11.7**	**50.5±13.2**	**52.5±11.9**	**56.0±13.0**	**.001**	**66.2±11.7**	**60.5±12.7**	**56.8±12.6**	**59.4±10.3**	**.045**
EDV rest (ml)	**84.7±37.9**	**107.8±54.4**	**93.5±31.9**	**86.7±31.7**	**.033**	**77.7±24.6**	**84.7±31.5**	**107.7±35.4**	**86.1±17.3**	**.007**
ESV rest (ml)	**36.8±27.9**	**57.4±50.6**	**43.5±22.4**	**37.6±24.0**	**.014**	**29.8±17.9**	**37.2±23.1**	**49.8±27.8**	**35.6±14.7**	**.015**
EDV post-stress (ml)	**85.7±35.9**	**109.0±51.5**	**111.1±53.2**	**91.6±31.3**	**.007**	**75.3±25.5**	**80.8±35.5**	**102.5±31.7**	**85.8±21.2**	**.023**
ESV post-stress (ml)	**37.4±26.2**	**58.9±49.1**	**57.3±43.4**	**43.2±25.3**	**.006**	**27.9±16.4**	**35.1±25.3**	**47.3±23.9**	**33.0±13.6**	**.018**
Diastolic blood pressure (DBP)										

Baseline (mmHg)	**81.7±13.3**	**75.9±11.1**	**76.0±14.5**	**79.3±11.5**	**.046**	80.1±11.0	78.8±12.3	81.8±11.8	85.3±10.0	.577
Peak (mmHg)	*77.8±15.2*	*74.4±11.3*	*72.2±14.2*	*72.5±10.0*	*.088*	86.3±20.9	82.7±13.4	94.1±19.0	96.9±28.4	.201
Systolic blood pressure (SBP)										
Baseline (mmHg)	**135.2±25.6**	**121.9±20.7**	**126.5±25.3**	**132.1±23.5**	**.024**	*131.1±18.9*	*120.9±16.5*	*135.4±19.7*	*135.3±10.5*	*.069*
Peak (mmHg)	143.8±27.7	141.3±26.0	137.3±24.6	132.8±27.9	.187	174.6±35.1	160.1±22.4	180.3±28.3	184.9±28.7	.139
Heart rate										
Baseline (bpm)	73.0±12.5	70.8±11.3	68.9±10.4	68.3±11.3	.134	72.1±13.3	78.7±16.2	75.6±13.1	74.0±13.5	.363
Peak (bpm)	**98.6±19.0**	**98.5±17.4**	**84.3±14.3**	**93.4±17.6**	**.001**	132.4±23.7	130.8±19.0	131.4±21.9	142.6±20.9	.658
HRR (%)	53.4±44.6	42.7±25.2	54.7±45.1	45.8±32.5	.410	46.2±34.5	55.1±41.5	44.0±31.7	65.0±55.7	.508

Data presented as mean ± standard deviation or number (%); CAD: coronary artery disease; CMD: coronary microvascular dysfunction; LVEF: left ventricular ejection fraction; EDV: end diastolic volume; ESV: end systolic volume; DBP: diastolic blood pressure; SBP: systolic blood pressure; HRR: heart rate response; [1]No ischemia /no cardiac history; [2]No ischemia/history of obstructive CAD; [3]Ischemia, obstructive CAD; [4]Ischemia, non-obstructive CAD
