# Supplementary material for: Psychological factors of suspect coronary microvascular dysfunction in patients undergoing SPECT imaging
Source: J Nucl Cardiol. 2020 Oct 6;29(2):768–78. doi: 10.1007/s12350-020-02360-5 (PMC8993740; doi:10.1007/s12350-020-02360-5)
Supplement: Supplementary file 2 — (PDF 686 kb) [file 12350_2020_2360_MOESM2_ESM.pdf]

Table S2. Acute emotions during cardiac stress testing stratified for groups and cardiac stress testing protocol.

	**Adenosine protocol (N = 201)**					**Exercise protocol (N = 88)**				
	Reference[1] (N=87)	History of CAD[2] (N=42)	Ischemic CAD[3] (N=35)	Suspect CMD[4] (N=37)	p-value	Reference[1] (N=44)	History of CAD[2] (N=20)	Ischemic CAD[3] (N=17)	Suspect CMD[4] (N=7)	p-value
Acute emotions during cardiac stress testing										
State anxiety score [mean]										
Anxiety baseline	2.84±3.0	2.68±2.34	2.73±2.20	3.01±3.04	.585	3.0±2.55	3.42±2.55	2.31±2.39	4.33±2.25	.599
Anxiety max. exertion	2.98±4.06	3.01±3.29	4.25±5.23	2.52±2.45	.125	3.59±4.61	2.57±2.02	2.04±2.18	4.80±5.89	.412
State sadness score [mean]										
Sadness baseline	9.51±7.92	7.83±7.36	10.19±6.93	10.27±6.22	.513	7.92±5.68	10.35±10.67	7.54±6.67	6.69±3.36	.607
Sadness max. exertion	2.98±4.06	3.01±3.29	4.25±5.23	2.52±2.45	.203	**8.29±7.88**	**19.22±24.76**	**9.20±7.84**	**18.91±19.10**	**.043**
Self-Reported tension/anxious	**24 (30%)**	**3 (8%)**	**3 (10%)**	**6 (19%)**	**.012**	*7 (19%)*	*0 (0%)*	*0 (0%)*	*1 (14%)*	*.065*

Data presented as mean ± standard deviation or number (%); CAD: coronary artery disease; CMD: coronary microvascular dysfunction; [1]No ischemia /no cardiac history; [2]No ischemia/history of obstructive CAD; [3]Ischemia, obstructive CAD; [4]Ischemia, non-obstructive CAD
