# Supplementary material for: Psychological factors of suspect coronary microvascular dysfunction in patients undergoing SPECT imaging
Source: J Nucl Cardiol. 2020 Oct 6;29(2):768–78. doi: 10.1007/s12350-020-02360-5 (PMC8993740; doi:10.1007/s12350-020-02360-5)
Supplement: Supplementary file 3 — (PPTX 194 kb) [file 12350_2020_2360_MOESM3_ESM.pptx]

## Slide 1
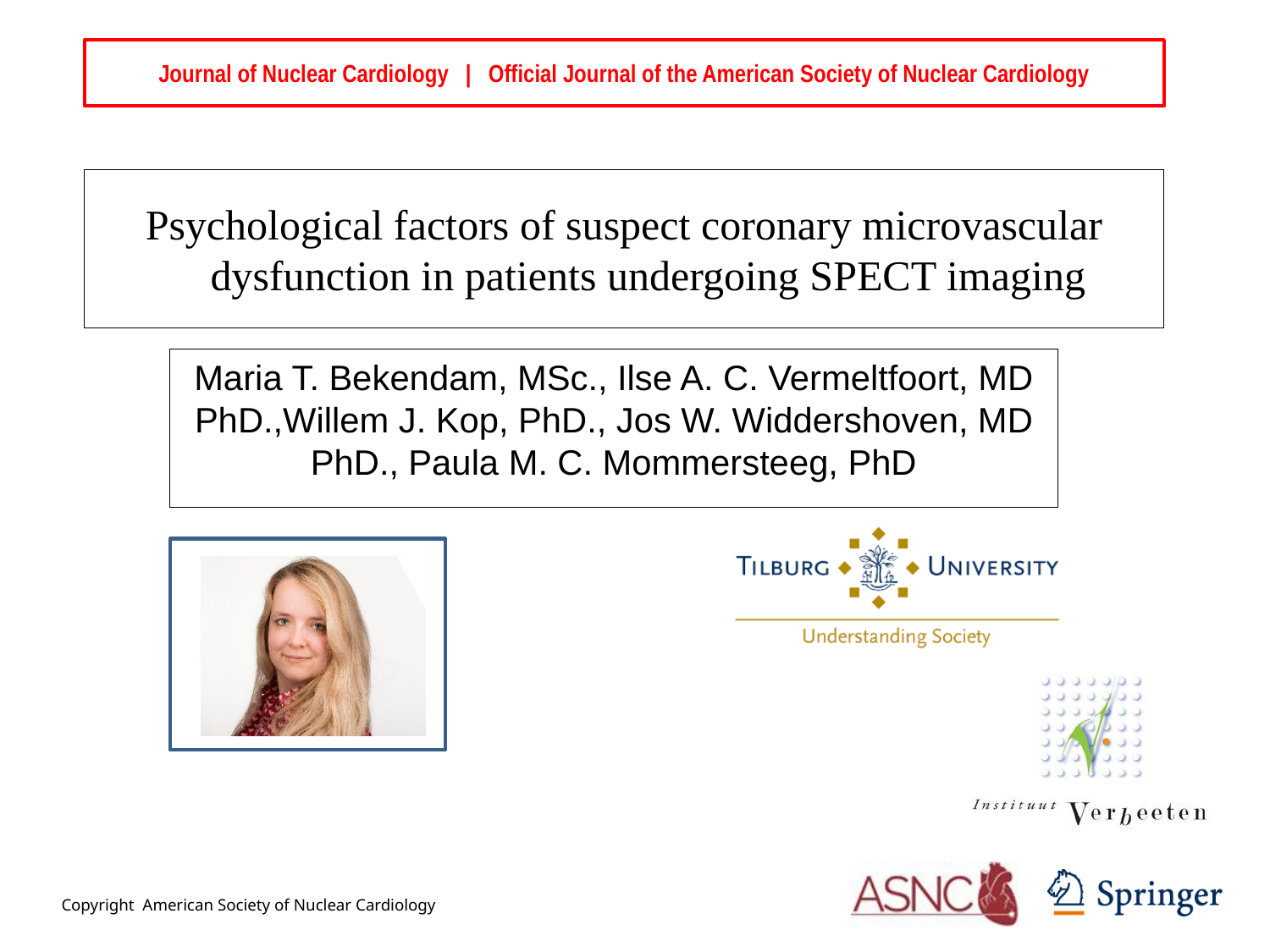

Journal of Nuclear Cardiology | Official Journal of the American Society of Nuclear Cardiology
# Psychological factors of suspect coronary microvascular dysfunction in patients undergoing SPECT imaging
Maria T. Bekendam, MSc., Ilse A. C. Vermeltfoort, MD PhD.,Willem J. Kop, PhD., Jos W. Widdershoven, MD PhD., Paula M. C. Mommersteeg, PhD
Copyright American Society of Nuclear Cardiology

## Slide 2
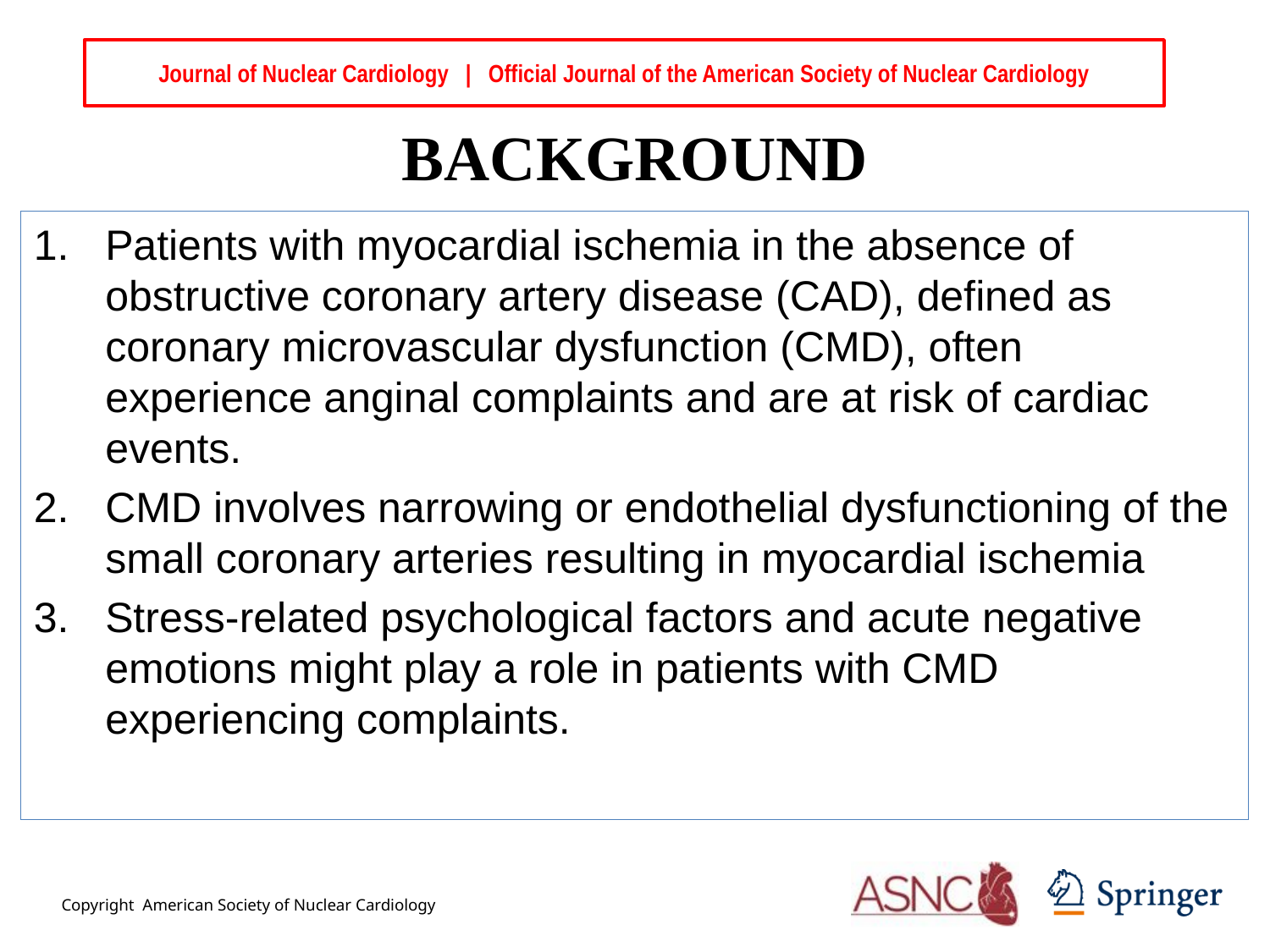

Journal of Nuclear Cardiology | Official Journal of the American Society of Nuclear Cardiology
# BACKGROUND
Patients with myocardial ischemia in the absence of obstructive coronary artery disease (CAD), defined as coronary microvascular dysfunction (CMD), often experience anginal complaints and are at risk of cardiac events.
CMD involves narrowing or endothelial dysfunctioning of the small coronary arteries resulting in myocardial ischemia
Stress-related psychological factors and acute negative emotions might play a role in patients with CMD experiencing complaints.
Copyright American Society of Nuclear Cardiology

## Slide 3
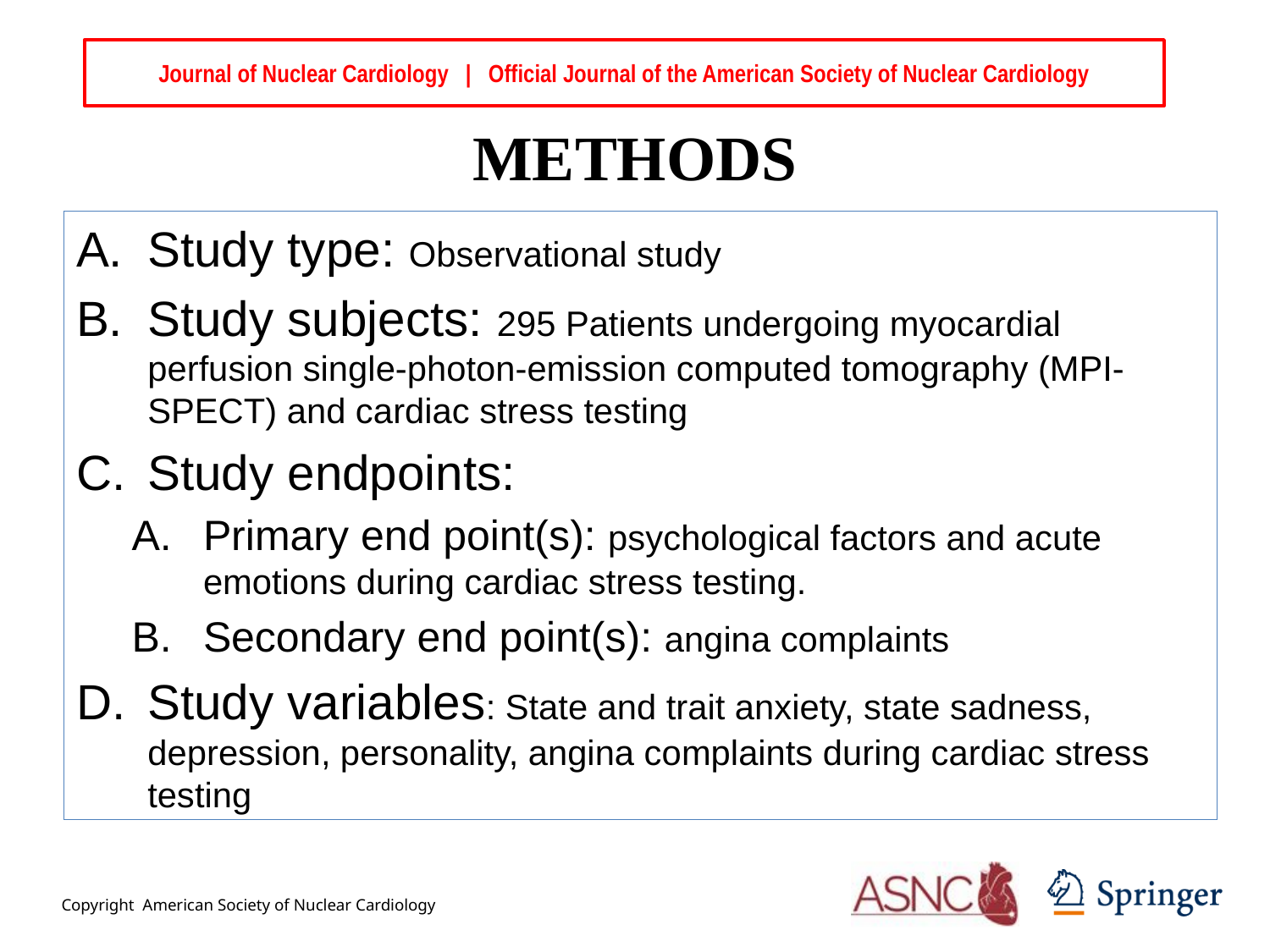

Journal of Nuclear Cardiology | Official Journal of the American Society of Nuclear Cardiology
# METHODS
Study type: Observational study
Study subjects: 295 Patients undergoing myocardial perfusion single-photon-emission computed tomography (MPI-SPECT) and cardiac stress testing
Study endpoints:
Primary end point(s): psychological factors and acute emotions during cardiac stress testing.
Secondary end point(s): angina complaints
Study variables: State and trait anxiety, state sadness, depression, personality, angina complaints during cardiac stress testing
Copyright American Society of Nuclear Cardiology

## Slide 4
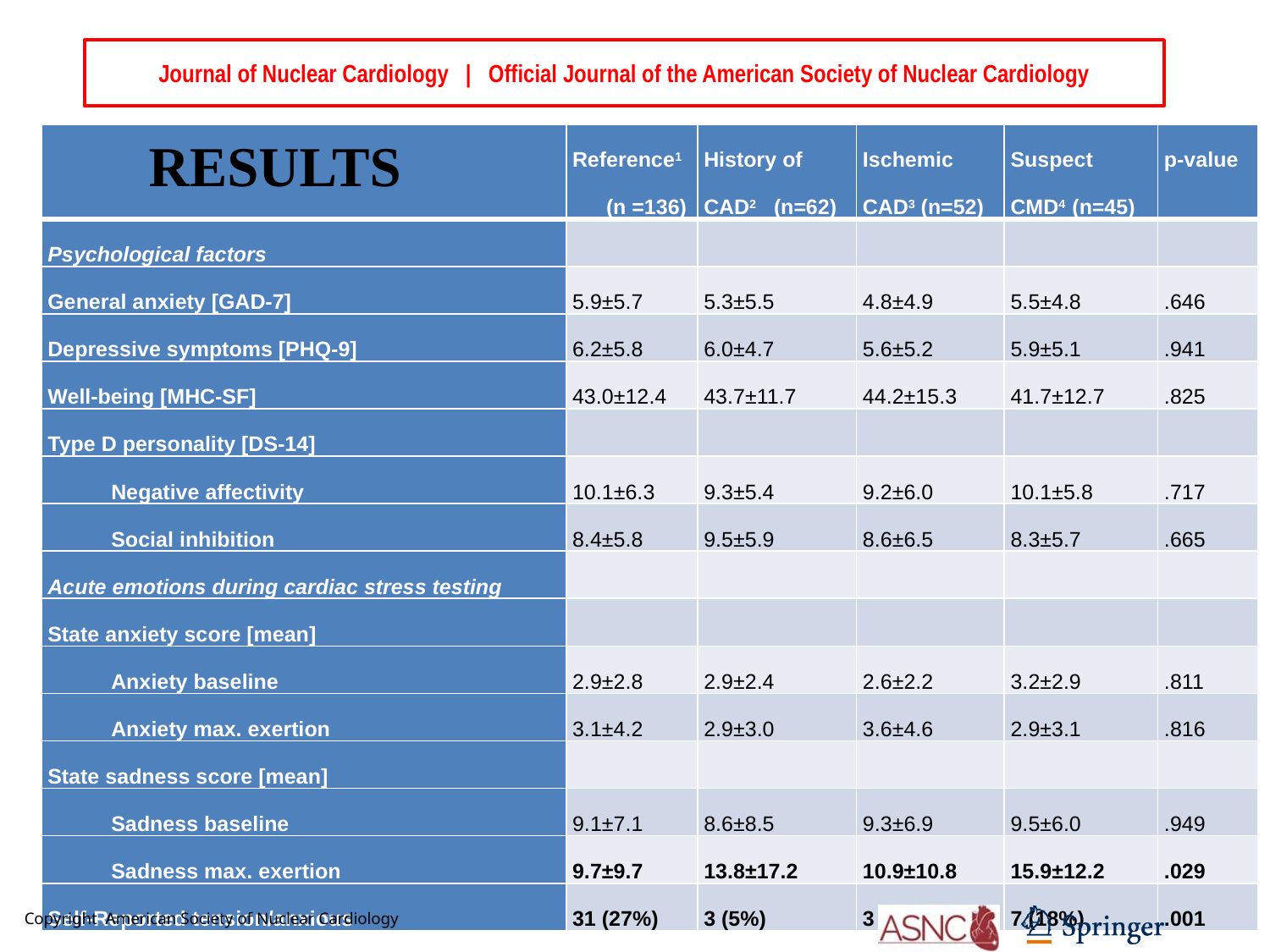

Journal of Nuclear Cardiology | Official Journal of the American Society of Nuclear Cardiology
# RESULTS
| | Reference1 (n =136) | History of CAD2 (n=62) | Ischemic CAD3 (n=52) | Suspect CMD4 (n=45) | p-value |
| --- | --- | --- | --- | --- | --- |
| Psychological factors | | | | | |
| General anxiety [GAD-7] | 5.9±5.7 | 5.3±5.5 | 4.8±4.9 | 5.5±4.8 | .646 |
| Depressive symptoms [PHQ-9] | 6.2±5.8 | 6.0±4.7 | 5.6±5.2 | 5.9±5.1 | .941 |
| Well-being [MHC-SF] | 43.0±12.4 | 43.7±11.7 | 44.2±15.3 | 41.7±12.7 | .825 |
| Type D personality [DS-14] | | | | | |
| Negative affectivity | 10.1±6.3 | 9.3±5.4 | 9.2±6.0 | 10.1±5.8 | .717 |
| Social inhibition | 8.4±5.8 | 9.5±5.9 | 8.6±6.5 | 8.3±5.7 | .665 |
| Acute emotions during cardiac stress testing | | | | | |
| State anxiety score [mean] | | | | | |
| Anxiety baseline | 2.9±2.8 | 2.9±2.4 | 2.6±2.2 | 3.2±2.9 | .811 |
| Anxiety max. exertion | 3.1±4.2 | 2.9±3.0 | 3.6±4.6 | 2.9±3.1 | .816 |
| State sadness score [mean] | | | | | |
| Sadness baseline | 9.1±7.1 | 8.6±8.5 | 9.3±6.9 | 9.5±6.0 | .949 |
| Sadness max. exertion | 9.7±9.7 | 13.8±17.2 | 10.9±10.8 | 15.9±12.2 | .029 |
| Self-Reported tension/anxious | 31 (27%) | 3 (5%) | 3 (6%) | 7 (18%) | .001 |
Copyright American Society of Nuclear Cardiology

## Slide 5
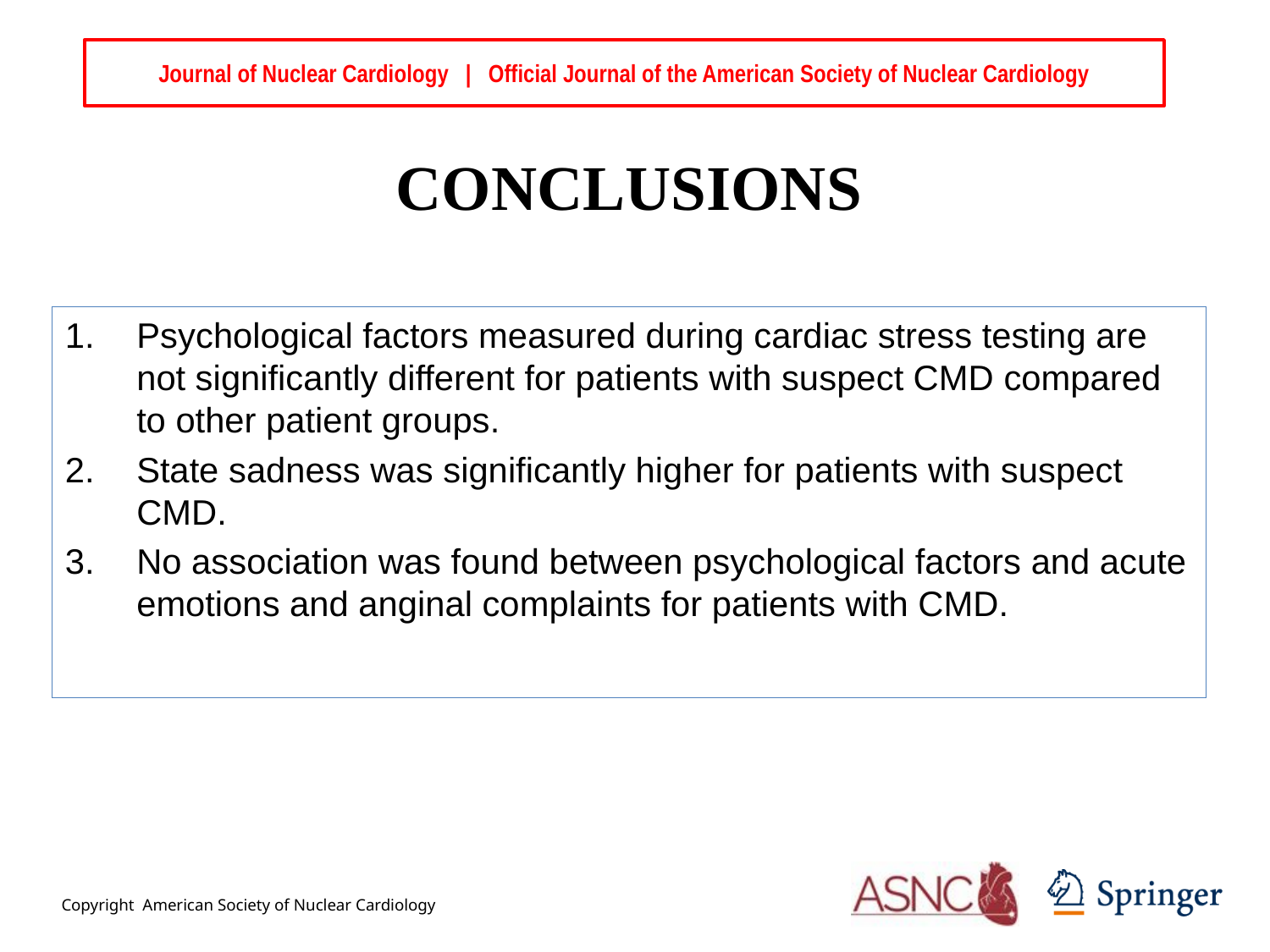

Journal of Nuclear Cardiology | Official Journal of the American Society of Nuclear Cardiology
# CONCLUSIONS
Psychological factors measured during cardiac stress testing are not significantly different for patients with suspect CMD compared to other patient groups.
State sadness was significantly higher for patients with suspect CMD.
No association was found between psychological factors and acute emotions and anginal complaints for patients with CMD.
Copyright American Society of Nuclear Cardiology
