# Supplementary material for: Psychological factors of suspect coronary microvascular dysfunction in patients undergoing SPECT imaging
Source: J Nucl Cardiol. 2020 Oct 6;29(2):768–78. doi: 10.1007/s12350-020-02360-5 (PMC8993740; doi:10.1007/s12350-020-02360-5)
Supplement: Supplementary file 4 — (DOCX 12 kb) [file 12350_2020_2360_MOESM4_ESM.docx]

Study by Bekendam et al. shows that coronary microvascular dysfunction (CMD) was not associated with more negative psychological trait factors compared to other myocardial ischemia groups. State sadness was higher for patients with CMD.
